# Supplementary material for: Plasma Nitrate and Nitrite Kinetics after Single Intake of Beetroot Juice in Adult Patients on Chronic Hemodialysis and in Healthy Volunteers: A Randomized, Single-Blind, Placebo-Controlled, Crossover Study
Source: Nutrients. 2022 Jun 15;14(12):2480. doi: 10.3390/nu14122480 (PMC9228981; doi:10.3390/nu14122480)
Supplement: Supplementary file 1 [file nutrients-14-02480-s001.zip › nutrients-1764475-supplementary.pdf]

**Plasma Nitrate and Nitrite Kinetics After Single Intake of Beetroot Juice in Adult Patients on Chronic Hemodialysis and in Healthy Volunteers: A Randomized, Single-Blind, Placebo-Controlled, Crossover Study**

Agustina Heredia-Martinez[1], Guillermo Rosa-Diez[1], Jorge R. Ferraris[1], Anna-Karin Sohlenius-Sternbeck[2], Carina Nihlen[3], Annika Olsson[3], Jon O. Lundberg[3], Eddie Weitzberg[3], Mattias Carlström[3*] and Rafael T. Krmar[3*]

[1] Department of Nephrology, Hospital Italiano de Buenos Aires, Buenos Aires, Argentina

[2] Department of Chemical and Pharmaceutical Safety, Research Institutes of Sweden (RISE), Södertälje, Sweden

[3] Department of Physiology and Pharmacology, Karolinska Institutet, Biomedicum 5B, Stockholm, Sweden

*Contributed equally to this work.

**Contact**:

Mattias Carlström, PharmD, PhD, Professor, Department of Physiology & Pharmacology, Karolinska Institutet, Biomedicum, 5B, Solnavägen 9, S-171 77, Stockholm, Sweden. Phone: +46 (0)8-524-86850. E-mail: mattias.carlstrom@ki.se

Rafael Tomas Krmar, MD, PhD, Department of Physiology and Pharmacology, Karolinska Institutet, Biomedicum, 5B, Solnavägen 9, SE-171 77, Stockholm, Sweden. Phone: +46 (0) 733200146. E-mail: rafael.krmar@ki.se

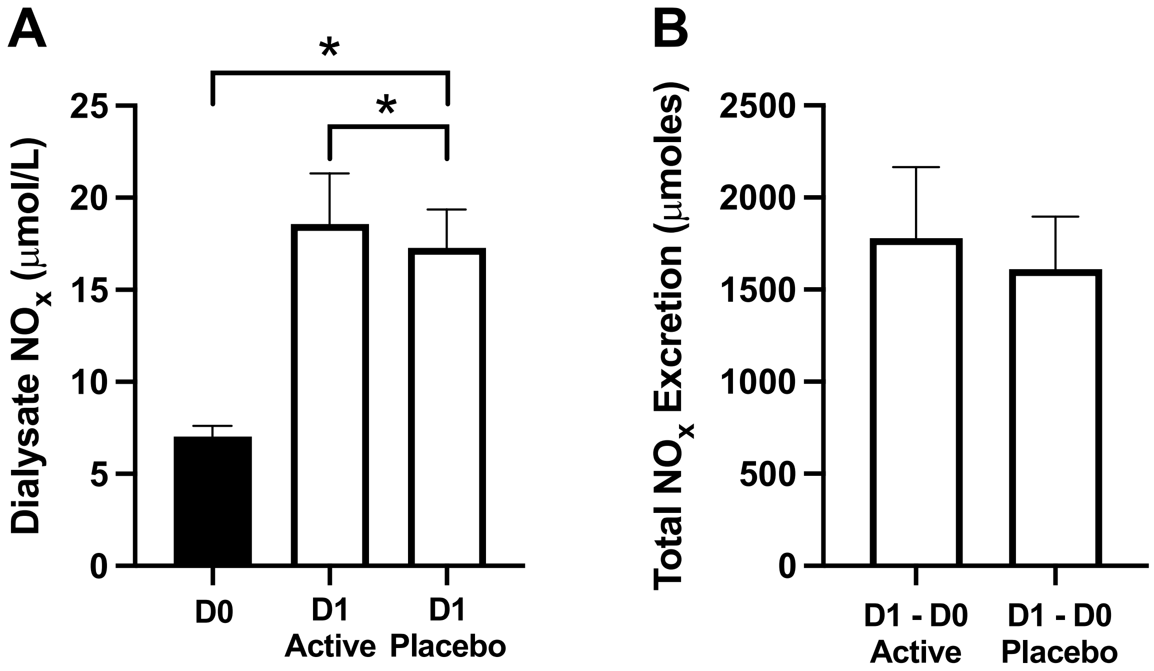


**Supplemental Figure S1.** *Dialysate and Excretion of Nitrate and Nitrite. Legend:* Levels of nitrate+nitrite (NOx) (Panel A) in fresh dialysate (D0) and from a representative sample of spent dialysate at the end of the hemodialysis session (D1) in hemodialysis patients prior to the intake of nitrate-rich (Active) or nitrate-depleted (Placebo) beetroot juice. Total amount of excreted NOx (Panel B) during the hemodialysis session prior to the intake of Active or Placebo juice. Values are presented as mean and standard deviation. $^{*}p < 0.05$ between indicated groups.

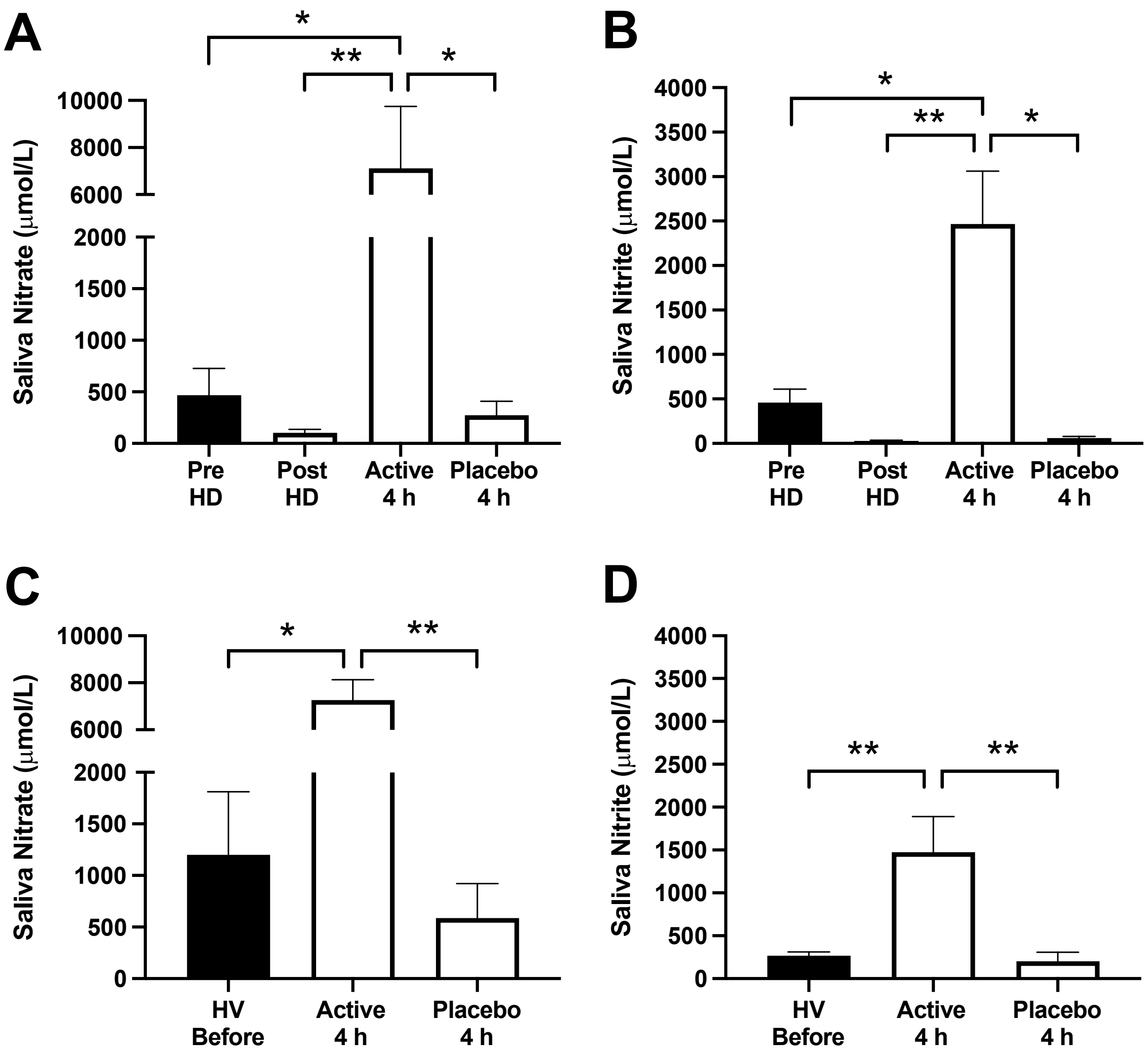


**Supplemental Figure S2**. *Saliva Nitrate and Nitrite Levels. Legend:* Saliva nitrate (A) and nitrite (B) levels in hemodialysis patients (HD) before and after dialysis and 4 hours after intake of nitrate-rich (Active) or nitrate-depleted (Placebo) beetroot juice. Saliva nitrate (C) and nitrite (D) levels in healthy volunteers (HV) before and 4 hours after intake of active juice or placebo juice. Values are presented as mean and standard deviation. $^{*}p < 0.05$; $^{**}p < 0.01$ between indicated groups.
